# [^68^Ga]ABY-028: an albumin-binding domain (ABD) protein-based imaging tracer for positron emission tomography (PET) studies of altered vascular permeability and predictions of albumin-drug conjugate transport

**DOI:** 10.1186/s13550-020-00694-2

**Published:** 2020-09-22

**Authors:** Emma Jussing, Li Lu, Jonas Grafström, Tetyana Tegnebratt, Fabian Arnberg, Helena Wållberg Rosik, Anders Wennborg, Staffan Holmin, Joachim Feldwisch, Sharon Stone-Elander

**Affiliations:** 1 Department of Clinical Neuroscience, Karolinska Institutet, SE17177, Stockholm, Sweden; 2 Department of Oncology and Pathology, Karolinska Institutet, SE17177, Stockholm, Sweden; 3 Department of Radiopharmacy, Karolinska University Hospital, SE17176, Stockholm, Sweden; 4 Comparative Medicine (KERIC), Karolinska University Hospital, SE17176, Stockholm, Sweden; 5 Department of Neuroradiology, Karolinska University Hospital, SE17176, Stockholm, Sweden; 6 Affibody AB, SE17165, Solna, Sweden

**Keywords:** Albumin-binding domain protein, Vascular permeability, Gallium-68, Positron emission tomography, Experimental tumor, Cerebral infarction

## Abstract

**Background:**

Albumin is commonly used as a carrier platform for drugs to extend their circulatory half-lives and influence their uptake into tissues that have altered permeability to the plasma protein. The albumin-binding domain (ABD) protein, which binds in vivo to serum albumin with high affinity, has proven to be a versatile scaffold for engineering biopharmaceuticals with a range of binding capabilities. In this study, the ABD protein equipped with a mal-DOTA chelator (denoted ABY-028) was radiolabeled with gallium-68 (^68^Ga). This novel radiotracer was then used together with positron emission tomography (PET) imaging to examine variations in the uptake of the ABD-albumin conjugate with variations in endothelial permeability.

**Results:**

ABY-028, produced by peptide synthesis in excellent purity and stored at − 20 °C, was stable for 24 months (end of study). [^68^Ga]ABY-028 could be obtained with labeling yields of > 80% and approximately 95% radiochemical purity. [^68^Ga]ABY-028 distributed in vivo with the plasma pool, with highest radioactivity in the heart ventricles and major vessels of the body, a gradual transport over time from the circulatory system into tissues and elimination via the kidneys. Early [^68^Ga]ABY-028 uptake differed in xenografts with different vascular properties: mean standard uptake values (SUV_mean_) were initially 5 times larger in FaDu than in A431 xenografts, but the difference decreased to 3 after 1 h. Cutaneously administered, vasoactive nitroglycerin increased radioactivity in the A431 xenografts. Heterogeneity in the levels and rates of increases of radioactivity uptake was observed in sub-regions of individual MMTV-PyMT mammary tumors and in FaDu xenografts. Higher uptake early after tracer administration could be observed in lower metabolic regions. Fluctuations in the increased permeability for the tracer across the blood-brain-barrier (BBB) direct after experimentally induced stroke were monitored by PET and the increased uptake was confirmed by ex vivo phosphorimaging.

**Conclusions:**

[^68^Ga]ABY-028 is a promising new tracer for visualization of changes in albumin uptake due to disease- and pharmacologically altered vascular permeability and their potential effects on the passive uptake of targeting therapeutics based on the ABD protein technology.

## Background

Dysfunctions and/or disruptions in endothelial permeability are key features of many human diseases such as inflammation, myocardial and cerebral infarction, tumors, and pathological angiogenesis. Increased vascular permeability together with impaired lymphatic drainage (enhanced permeability and retention (EPR)) of diseased tissues are key characteristics that are taken advantage of to improve depositions of macromolecular drugs [1]. Imaging alterations in vascular permeability can be useful for characterizing/diagnosing the diseased tissues. Furthermore, if the imaging utilizes signal-producing molecules that are structurally related to drugs (theranostics), information from the diagnostic studies might also be useful for predicting and optimizing therapy. However, to make assessments of and predictions about targeting abilities and therapeutic actions, the relative amounts of specific and non-specific uptakes of the theranostics should be estimated. There are at least three strategies used for estimating specificity. *One*, the tracer uptake in “positive” models is compared with uptake in “negative” models lacking the target. This approach requires that all other parameters in the models (such as vascular permeability and architecture, necrosis, interstitial pressure, etc.) are essentially the same. *Two*, pre-blocking or displacement experiments are performed to demonstrate specific uptake. Total blocking of the binding sites can, however, be difficult to achieve due either to pharmacological difficulties in administering large enough doses, to differences between tissues if the blocking and non-blocking experiments are not performed in the same animal or to rapidly changing tissue characteristics when the experiments are performed on the same animals but not close enough in time. *Three*, studies of the targeting agent are compared with those of a non-binding size-matched control. Based on imaging studies with ^89^Zr-DFO-albumin, it has been argued that this third approach should be required when evaluating the binding capability of targeting agents as large as or larger than 65 kDa albumin [2] and this approach has also been beneficial for smaller macromolecules such as annexin (35 kDa) [3, 4]. To perform paired studies in the same animal, the control must be labeled with an appropriate short-lived radionuclide so the targeting/non-targeting uptakes can be evaluated as close in time as possible. If in different animals, the same concerns as in the first two strategies must be considered.

Albumin, the most abundant plasma protein, has been frequently used as a carrier platform to deposit drugs [5–8] and diagnostic imaging agents [9–13] in tissues with altered vascular permeability. Albumin’s circulatory half-life is quite long, approximately 20 days, due to its binding to the neonatal Fc receptor (FcRn) [14]. Albumin is transported through the normal endothelium by transcellular pathways [15] at a rate of approximately 5% per hour [16], but the transport pathways change and the rates of and total albumin uptake often increase when endothelial barriers are disrupted. Signal-producing probes and/or drugs that are tightly associated, conjugated, or fused with albumin should be kept in circulation and be delivered to tissues at a rate and in a manner similar to that of serum albumin. Once transported, the intracellular environment may alter the albumin-drug/probe bonds and albumin can be catabolized. The intracellular properties of the albumin-binding molecule itself may then play an increasingly important role on the total retention. Uptake patterns for different albumin-bound agents can consequently differ over time (compare, for example, that of an albumin-binding folate conjugate [17] with that of albumin-binding truncated Evans blue [18]). To examine the specificity of a drug that uses albumin as a carrier, a non-targeting control should be as similar as possible to the targeting drug. The uptake of the control in tissues at early times should reflect the properties of the albumin carrier and as time progresses that of the drug/diagnostic and the degree to which it is still bound to albumin.

**Fig. 1:**
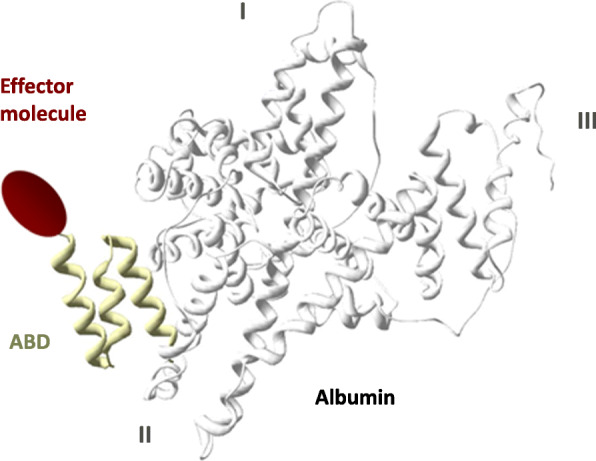
The Albumod technology (schematic model). The Affibody albumin-binding domain (ABD) protein (yellow) binds to albumin (gray/white, crystal structure, PDB no: 1TF0) via a regioselective, tight interaction with domain II as earlier demonstrated by Johansson [20] and based on crystal structure by Lejon [21]. Effector molecules (red) are site-specifically attached to ABD in a position remote from the albumin binding surface. The effector molecule is thus held in circulation with a biological half-life similar to that of albumin, thereby increasing the probability that the administered effector molecule will reach (and interact) with its target. For radiodiagnostics, the effector is a radionuclide. For therapeutics, the effector may be a therapeutic radionuclide, targeting drugs, particles, or fusion proteins

A small (5.1 kDa) albumin-binding domain (ABD) protein folded into a three-helix bundle has been engineered to bind to serum albumin with very high affinity [19]. The albumin-binding surface is located on one face of the bundle, involving surface exposed side chains of amino acid residues in helices two and three. ABD bound to albumin does not interfere with the binding of the latter to FcRn. Extended circulation half-lives have been demonstrated for a number of biopharmaceuticals fused to ABD (and consequently bound to albumin) (Albumod™ technology, Affibody AB, Fig. 1) that target peripheral diseases [22–34] as well as diseases of the central nervous system [35, 36]. Improved therapeutic efficacy related to the improved availability (longer circulation half-lives) has also been demonstrated for ABD fused Affibody molecules loaded with radioactive lutetium-177 [31] and with an immunotoxin [37], but not when coupled to Trail [38]. In this study, the Albumod™ technology was utilized to develop a novel radiotracer for in vivo imaging of the distribution and uptake of albumin carrying a non-therapeutic ABD-based control. To enable site-specific radiolabeling, 1,4,7,10-tetraazacyclododecane-1,4,7-tris-acetic acid-10-maleimidoethylacetamide (mal-DOTA) was coupled to a single cysteine in ABD. The resulting molecule is denoted ABY-028. The mal-DOTA in ABY-028 was then chelated with the positron-emitting radionuclide gallium-68 (^68^Ga) to take advantage of the non-invasiveness, the spatial resolution, sensitivity, and quantification capability of positron emission tomography (PET) imaging. The labeled ABD ([^68^Ga]ABY-028) binds in vivo to circulating albumin immediately after intravenous (i.v.) administration. In addition to developing labeling conditions, the in vivo distribution of the albumin-bound [^68^Ga]ABY-028 was examined to validate that the radiotracer did indeed behave as an albumin-carried probe. Distribution was examined first in healthy rodents and subsequently studies were performed that address some common considerations when utilizing this and similar-sized or larger macromolecular theranostics: how the probe localizes in tissues of heterogeneous permeability was examined here in tumor models with different vascular characteristics and how/if uptake would be sensitive to acute variations in permeability was examined here by pharmacological manipulation in a tumor model and after an acute blood-brain-barrier (BBB) disruption in a model of induced stroke [39].

## Methods

### General

All chemicals used in the synthesis, purification, and analyses of [^68^Ga]ABY-028 were commercially available and of analytical grade unless otherwise specified. [2-^18^F]-2-Fluoro-2-deoxy-D-glucose ([^18^F]FDG) was obtained from batches produced daily in house for clinical PET. Radiochemical analyses were performed by high-performance liquid chromatography (HPLC) and instant thin layer chromatography (iTLC). Ultraviolet- (210 nm) and radio-detectors in series were used for radio-HPLC. Size exclusion chromatography (SEC) was performed on a Superdex Peptide 10/300 GL column (General Electric Healthcare Life Sciences) eluted with phosphate-buffered saline pH 7.4 (PBS), flow 1.0 mL/min. Reversed phase chromatography (RPC) was performed on a C18 218TP 5 μm column (Vydac) eluted with a gradient (0➔8 min B 0➔80%, 8➔9 min B 80➔100%, 9➔13 min B 100➔0%), flow 1.0 mL/min, mobile phase A: 0.1% trifluoroacetic acid (TFA) in acetonitrile: water (CH_3_CN:H_2_O) 10:90, mobile phase B: 0.1% TFA in CH_3_CN:H_2_O 60:40). iTLC was performed using iTLC-paper impregnated with silica gel for chromatography (Varian), mobile phase ammonium acetate (1 M): methanol = 1:1 (without or with ethylenediaminetetraacetic acid (EDTA) added), and analyzed with a Bioscan AR-2000 TLC scanner. Phosphorimaging plates were scanned using a Typhoon FLA 7000 (GE Healthcare).

### Production of ABY-028

ABY-028 was produced by standard solid phase peptide synthesis (SPPS) including synthesis of the ABD peptide, cleavage and purification of the crude peptide, coupling of mal-DOTA, additional purification followed by microfiltration and lyophilization (Bachem AG, Switzerland). The starting materials were either manufactured by Bachem AG or obtained commercially from qualified suppliers and were fully tested against defined specifications using standard test methods. Synthesis of the ABD peptide was performed in an automated peptide synthesizer and consisted of alternating coupling, acetylation, and Fmoc-deprotecting procedures. The coupling was performed in dimethylformamide (DMF) or DMF/dichloromethane (DCM) as solvent. It consisted of coupling the N-α-protected amino acid derivatives to the preceding amino acid in the presence of an activating reagent and a base, if necessary. To avoid the formation of deletion sequences as by-products, a systematic acetylation procedure (capping) was performed after the coupling step using acetic anhydride, rendering unreacted coupling sites inactive. After each capping step, the resin was washed with DMF, followed by cleavage of the Fmoc-group with piperidine in DMF and subsequent washing with DMF and isopropyl alcohol (IPA). The SPPS was completed by washing the peptide resin with DMF and IPA and subsequently drying. Cleavage of the peptide from the resin and concomitant cleavage of the side-chain protecting groups was accomplished by treatment of the peptide resin with TFA. The resin was filtered off and washed with TFA. The peptide product was then precipitated and washed in cold diisopropyl ether and dried under reduced pressure. The crude peptide was purified by preparative RPC and pooled fractions with adequate purity were lyophilized. The lyophilized ABD peptide was dissolved in 10% (V/V) acetic acid and loaded onto an ion exchange column (acetate form). The elution was performed with 10% (V/V) acetic acid. The peak fraction was collected and filtered through a 0.2-μm membrane filter and lyophilized. The purified ABD peptide was coupled with mal-DOTA in ammonium acetate buffer/DMF and the resulting product, ABY-028, was then purified by preparative RPC using acetonitrile gradient elution. The collected main fractions were checked by analytical HPLC, pooled accordingly, and lyophilized. The lyophilized ABY-028 was dissolved in H_2_O, filtered through a 0.2-μm membrane filter and lyophilized, yielding a white to off-white powder (drug substance, DS). The pharmaceutical formulation solution (drug product, DP) was prepared by dissolving 220 μg/mL (net peptide) of ABY-028 DS in 50 mM sodium acetate buffer pH 5.3 with 2% (m/m) D-mannitol, pre-filtration for bioburden reduction and sterile filtration through two membrane filters (0.2 μm). The collected filtrate was filled aseptically into quartz-coated class I plus vials (fill volume 0.5 mL). Subsequently, the vials were closed with lyophilization rubber stoppers and transferred into the freeze dryer. The lyophilized peptide was stored at − 20 °C.

### Radiochemistry

^68^Ga (half-life 68 min; β^+^ 89%; E_β+_ max. 1.9 MeV) was obtained from a ^68^Ge/^68^Ga-generator (Eckert & Ziegler, IGG-100 or -101). The ^68^Ga in the eluate was obtained in a more concentrated solution by using Chromafix 30-PS-HCO_3_/HCl [40], Bond Elut-SCX/sodium chloride (NaCl) [41], or by fractionation [42], the latter either manually or by using an automated synthesis module (Eckert & Ziegler, PharmTracer). Hydrochloric acid (Rotem, HCl sterile solution, 0.1 N) was used as the eluent and the fraction used of the maximum ^68^Ga-containing eluent was 1–2.5 mL, depending on the amount of radioactivity desired.

Labeling was manually performed in the final procedure using the following protocol: The collected ^68^Ga eluate was adjusted to pH 4.0 using sodium acetate (2 M, pH 4.6) and added, together with ethanol (EtOH) (200 μL, 99%, Kemetyl AB) to inhibit radiolysis, to the ABY-028 (110 μg freeze-dried, 19.5 nmol, Affibody AB) in a quartz-coated vial. The resulting solution was heated at 75 °C for 15 min. Unreacted or partially complexed ^68^Ga was chelated by adding EDTA (0.1 M, 25 μL) for 5 min while the solution cooled to room temperature (RT) with venting to reduce the EtOH. Water (Millipore filtered, 18 MΩ, 4 mL) was added and the reaction solution was eluted through a solid phase extraction (SPE) cartridge (Waters, Oasis HLB Light (30 mg), pre-conditioned with EtOH (1 mL) then H_2_O (2 mL)). The SPE was then washed with H_2_O (Millipore filtered, 18 MΩ, 4 mL). The ^68^Ga-labeled product was eluted from the SPE using sterile EtOH (50%), here separated in 0.1 mL fractions for the intended animal studies. Immediately prior to the in vivo studies, an aliquot of the maximum product-containing fraction(s) was diluted with sterile PBS or NaCl (0.9%) to a maximum EtOH concentration of 7.5%. The product obtained was analyzed by radio-iTLC (Rf: 0, 0.3 and 1.0 for ^68^Ga-colloid, [^68^Ga]ABY-028 and ^68^Ga^+3^/^68^Ga-EDTA, respectively) and/or by radio-HPLC (retention times 10.3 and 16.8 min (SEC) or 9.2 and 2.6 min (RPC) for [^68^Ga]ABY-028 and low molecular weight ^68^Ga^+3^, respectively).

### Binding assays

ELISA plates were coated overnight with human serum albumin (HSA), 1 μg/mL in carbonate buffer (100 mM Na_2_CO_3_/NaHCO_3_ pH 9.6), 50 μL/well. The plates were blocked by incubation with 100 μL 5% non-fat dry milk (NFDM) in PBST (1× PBS/0.05% Tween) for > 2 h at RT under slight shaking and then washed two times with 200 μL PBST.

[^68^Ga]ABY-028 was added in concentrations ranging approximately from 2 fM to 0.1 μM in PBST, 50 μL/well, and incubated for 1 h in RT under slight shaking. Plates were washed four times with 200 μL PBST/well. The ELISA plates exposed a phosphorimaging plate overnight, which was then scanned using a phosphorimager. Regions of interest (ROIs) were drawn around the areas corresponding to the wells of the plate. The amount of radioactivity in the different wells was quantified, background subtracted, and normalized by scaling between 0 and 1. GraphPad Prism 5 was used to estimate the equilibrium binding constant (Kd). The concentration of [^68^Ga]ABY-028 used for the dilution series was determined by A_280_ measurement and concentrations were adjusted accordingly. The mean Kd was calculated as the mean from three separate experiments.

To analyze binding specificity, an ELISA plate was coated with HSA, blocked with NFDM and washed as described above. To six wells, 50 μL [^68^Ga]ABY-028 in PBST was added to a final concentration of 0.1 μM. To three of the wells, non-labeled ABY-028 was added in advance, final concentration 20 μM, i.e., 200-fold excess of non-labeled ABY-028 over labeled. Plates were incubated with shaking at RT for 1 h, washed, exposed, and analyzed as described above.

### Tumor cell lines and animals

A431 (human epidermoid carcinoma) and FaDu (human squamous carcinoma of the hypopharynx) cell lines were purchased from American Type Culture Collection (ATCC). The cells were cultured in Dulbecco’s Modified Eagle’s Medium (4500 mg/L D-glucose containing L-glutamine) and were maintained at 37 °C in a humidified atmosphere, containing 5% CO_2_. All media were additionally supplemented with 1 mM sodium pyruvate, 100 units penicillin/mL, 100 μg streptomycin/mL, and 10% fetal bovine serum (Gibco). Media for FaDu cells were further supplemented with 0.1 mM non-essential amino acids and 2 mM HEPES. The integrity of the cell lines was verified with short tandem repeat profiling cell authentication analysis (LGC Standards).

Rats (NIH-Foxn1mu and Sprague-Dawley) and mice (severe combined immunodeficiency (SCID)) were purchased from Charles River (Sulzfeld, Germany) and MMTV-PyMT transgenic mice were obtained from the breeding facility at the Wallenberg laboratory animal facility (Karolinska Institutet, Stockholm, Sweden). All animals were housed under standard conditions according to local regulations, with access to food and water ad libitum in the Department of Comparative Medicine at Karolinska University Hospital, Solna, and/or at the Wallenberg laboratory at Karolinska Institutet, Solna.

### PET imaging, data collection, and processing

Rodents were anaesthetized (1.5% isoflurane (Virbac, Carros Cedex, France) blended with air (7:3) in a vaporizer (E-Z systems) delivered through a Microflex non-rebreather mask (Euthanex Corp)) and then i.v. injected (tail vein) with the radiotracer. PET imaging was performed using a microPET Focus 120 (CTI Concorde Microsystems, Knoxville, TN, USA). Data were acquired in list mode and reconstructed using Filtered Back Projection for the constant bed motion (CBM) scans or, to increase the spatial resolution in the dynamic scans, Ordered Subset Estimation Maximum in 2 dimensions, 4 iterations, and 16 subsets. Data, normalized and corrected for randoms, dead time, and radioactivity decay, were processed using MicroPET Manager and evaluated using the Inveon Research Workplace (IRW) software (Siemens Medical Systems, Malvern, PA, USA). The ROI selections were based on the PET images and the radioactivity in ROIs was calculated as mean or maximum standard uptake values (SUV_mean_ or SUV_max_) normalized to body weight [43]. Blood ROIs were delineated on the left ventricle of the heart in the first frames of the scan. Liver ROIs were drawn on one lobe. Muscle ROIs were drawn on the front (rat) and hind (mouse) leg muscle on the images summed over the entire scan. Tumor ROIs were delineated on images summed over the last 15–30 min of the scan and, where applicable, thresholded 75%, based on palpation measurements. Line profile graphs were generated for rulers drawn manually in the IRW image view window over the tumor areas of interest. Infarcted and contralateral brain ROIs were delineated on images summed over the entire scan. Growth of the tumor xenografts was monitored by caliper measurements.

Biodistribution studies were performed in ten animals (5 mice and 5 rats, single measurements). Radiotracer uptake was examined initially in single scans of mice xenograft models (3 lung cancer (NCI-H2122), 2 prostate cancer (22Rv1), 1 breast cancer (MDA-MB231), and 7 head and neck cancer (FaDu)) but subsequently in a mouse with MMTV-PyMT mammary tumors (1 with multiple tumors) and in rat xenograft models (4x2 bilateral FaDu and 1x2 bilateral A431). Study-specific details were as follows.

#### For the [^68^Ga]ABY-028 biodistribution studies

Several studies with different scanning protocols were performed. Three representative studies are shown here. In a whole body CBM study, [^68^Ga]ABY-028 (26.8 MBq, 1 mL) was i.v. injected in a Sprague-Dawley rat (male, 560 g) and scanned for 45 min. In a fixed bed dynamic scan, [^68^Ga]ABY-028 (14.6 MBq, 1 mL) was i.v. injected in a NIH-Foxn1mu rat (male, 312 g), scanned 0–30 min (thorax) and, after moving the bed position, 34–64 min (head). In a whole body dynamic scan, [^68^Ga]ABY-028 (10 MBq, < 0.2 mL) was i.v. injected in a SCID mouse (male, 29 g) and scanned for 60 min.

#### For the single and dual tracer studies in tumor-bearing animals

[^68^Ga]ABY-028 in bilateral FaDu xenografts: FaDu cells were implanted subcutaneously (s.c.) on the same day with 5 × 10^6^ and 2 × 10^6^ cells on the left and right shoulders, respectively, of a NIH-Foxn1mu rat (male, 417 g). PET imaging was performed 13 days after the inoculations (tumor size of 7 × 14 mm (left, T_1_, Fig. 4a) and 7 × 11 mm (right, T_2_, Fig. 4a)). [^68^Ga]ABY-028 (81.5 MBq, 1 mL) was i.v. injected in a tail vein and data were acquired for 180 min (only the first 60 min shown here in the comparison with A431).

[^68^Ga]ABY-028 in bilateral A431 xenografts: Double s.c. inoculations (same day) were performed with 5 × 10^6^ human epidermoid carcinoma (A431) cells on the left and the right shoulder of a NIH-Foxn1mu rat (male, 336 g). PET imaging was performed 16 days after inoculations (tumor size of 7 × 8 mm (left, T_3_, Fig. 4b) and 4 × 17 mm (right, T_4_, Fig. 4b)). [^68^Ga]ABY-028 (27.9 MBq, 1 mL) was i.v. injected in a tail vein and data were acquired for 60 min. Without moving from the camera, nitroglycerin ointment (NG, glyceryltrinitrate, Rectogesic®, ProStrakan Ltd, UK) (1 mg/tumor), was cutaneously applied over the tumors and data were then acquired for an additional 120 min.

[^68^Ga]ABY-028 and [^18^F]FDG in the MMTV-PyMT transgenic mouse model: A 12-week-old mouse (female, 31 g) was i.v. injected with [^18^F]FDG (7.8 MBq, < 0.2 mL) and scanned for 60 min and, on next day, with [^68^Ga]ABY-028 (14.9 MBq, < 0.2 mL) and scanned for 120 min. The mouse had fasted 5 h prior to the [^18^F]FDG injection.

[^68^Ga]ABY-028 and [^18^F]FDG in FaDu xenografts: Double s.c. inoculations (same day) were performed with 5 × 10^6^ cells on the right shoulder and on the left mid-back of a NIH-Foxn1mu rat (male, 252 g). On the following days after inoculation: on day 13, [^18^F]FDG (31.1 MBq, 1 mL) was i.v. injected and data collected for 160 min; on day 14, [^68^Ga]ABY-028 (53.3 MBq, 1 mL) was i.v. injected and data collected for 195 min; on day 15, [^18^F]FDG (34.4 MBq, 1 mL) was i.v. injected and data collected for 160 min; and on day 16, [^68^Ga]ABY-028 (45.8 MBq, 1 mL) was i.v. injected and data collected for 195 min. The rat had fasted 5 h prior to the [^18^F]FDG injections. Caliper measurements of the tumors were L: 9 × 10 mm, R: 9 × 10 mm on day #13 and L: 6 × 11 mm, R: 9 × 12 mm on day #16.

#### For the [^68^Ga]ABY-028 study during acute cerebral infarction

One male Sprague-Dawley rat (409 g) was subjected to 90 min occlusion of the M2 segment of the middle cerebral artery (MCA) using a previously described procedure [39]. Briefly, anesthesia was induced at a 4% and sustained at a 2% isoflurane concentration in an air to oxygen mixture (7:3). A 0.007-inch (0.18 mm) microwire (Hybrid; Balt Extrusion, Montmorency, France), sheeted inside a 0.020-inch (0.51 mm) microcatheter (Ultraflow; Covidien, Mansfield, MA, USA), was introduced through the ventral tail artery and positioned in the M2 segment of the MCA under X-ray fluoroscopy guidance. The animal was placed in the PET scanner with the brain in the field of view within five min after placement of the microwire in the MCA. [^68^Ga]ABY-028 was administered via the tail vein (58 MBq, 0.7 mL). Data were acquired continuously during 80 min from the injection.

After the rat was sacrificed at the end of the PET scan, the brain was immediately removed and frozen in dry ice and sectioned using a cryomicrotome (CM 3050S, Leica Microsystems, Wetzlar, Germany) with a thickness of 30 μm. The sections were exposed on phosphorimaging plates that were subsequently scanned using a phosphorimager.

For anatomical correlates for the uptake, T2-weighted fast spin-echo 3D high-field (9.4 T) magnetic resonance images were acquired from one naive male Sprague-Dawley rat and co-registered using the IRW software. ROIs were manually traced for the brain region that showed increased uptake in the occluded MCA territory and for the corresponding region in the contralateral hemisphere.

## Results

### Peptide synthesis

Three batches of ABY-028 were produced by standard peptide synthesis, one small-scale test batch (30 mg product), one technical batch (15 g product), and one good manufacturing practice (GMP) batch (8.8 g product). The technical batch yielded ABY-028 with a purity of 92.4% and was used for preliminary labeling and pilot preclinical studies and a toxicity study. The GMP batch yielded ABY-028 drug substance with a purity of 98.9% that was used for filling of DP vials. The DP vials were used for optimization and validation of the ^68^Ga-labeling method, preclinical studies, and stability studies. The stability study proved that the drug product ABY-028 with 110 μg/vial (lyophilizate) is stable when stored at − 20 °C for 24 months (end of stability study). The purity and related substance as determined by analytical HPLC remained constant (range 96.8 to 98.0%), the H_2_O content varied between 0.7 and 1.2% (release 1.1%), the amount per vials remained constant (range 105.7 to 111.8 μg/vial), absence of aggregates (with exception of one with 0.2% area at the 18 months pull point), and retained potency (albumin binding). All related substances detected in the DS are well below the identification threshold of 0.5% for organic impurities in peptides obtained by chemical synthesis (Ph. Eur. General Monograph 07/2009:2034, Substances for Pharmaceutical Use).

### Radiotracer preparation and characterization

**Fig. 2:**
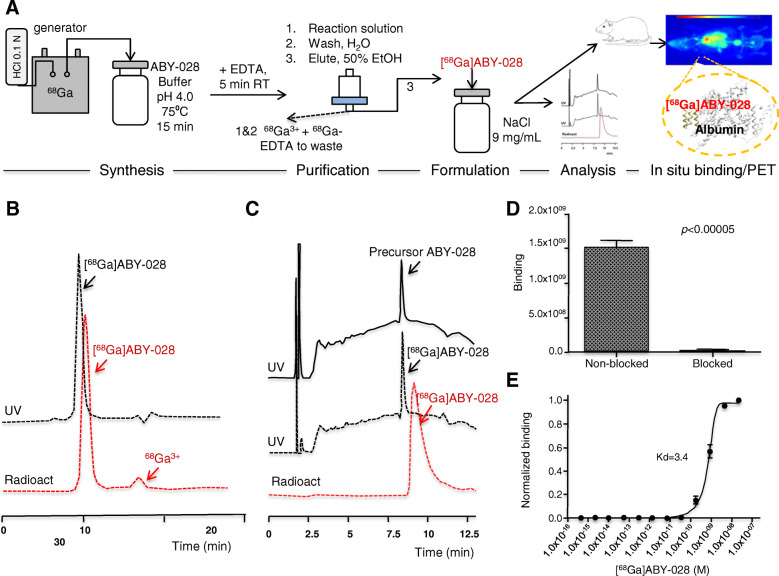
Radiolabeling and analyses of [^68^Ga]ABY-028. **a** The major steps in the radiolabeling with ^68^Ga. **b** Size-exclusion chromatography of the reaction solution after the labeling of [^68^Ga]ABY-028 (UV and radio). **c** Reversed phase chromatography of reference precursor ABY-028 (UV) compared with the purified [^68^Ga]ABY-028 (UV and radio). **d** Analysis of [^68^Ga]ABY-028 binding to immobilized HSA with and without blocking using a 200-fold excess of non-labeled ABY-028. **e** Analysis of [^68^Ga]ABY-028 binding to immobilized HSA. The normalized radioactivity is plotted against the concentration of [^68^Ga]ABY-028

The scheme for the labeling procedure is shown in Fig. 2a. Radiolabeling with ^68^Ga proceeded essentially as most literature procedures with DOTA-modified polypeptides. For best yields, heating at 75–85 °C and reaction times of 10–15 min were required (ABY-028 tolerates heating up to 90 °C). Of the three methods tested to concentrate the ^68^Ga used for radiolabeling, fractionation worked best. Approximately 70% of the eluted ^68^Ga could be collected in a 1-mL fraction and no additional work-up of the eluate was required as long as the generator was in regular use to keep levels of competing metal impurities at a minimum [40]. High radiolabeling conversions were also obtained with the Chromafix-purified ^68^Ga, but overall yields were lower due to radioactivity losses in the concentration step. ABY-028 did not tolerate the NaCl concentrations in the Bond Elut-SCX/NaCl method—essentially, no labeled product was obtained. The first pilot PET studies occasionally showed appreciable renal eliminations of radioactivity at early times. Consequently, to chelate and remove possible absorbed and/or loosely bound/chelated ^68^Ga, EDTA was added to the reaction mixture post-labeling but prior to the SPE purification. Radiochemical conversions of more than 80% were obtained. The radiochemical purity analyzed by radio-HPLC and radio-iTLC was 95 ± 3%. Examples of radio-HPLC analyses are shown in Fig. 2b, c.

### Binding assay results

The in vitro specificity study showed that [^68^Ga]ABY-028 bound with high specificity to immobilized HSA. Pre-saturation of the HSA binding sites with a 200-fold excess of non-labeled ABY-028 significantly reduced the radioactivity concentration (*p* value <5 × 10^−4^), thus indicating specific binding to albumin (Fig. 2d). The assay thus confirmed that binding to immobilized HSA was preserved after labeling. The equilibrium binding constant for binding of [^68^Ga]ABY-028 to HSA was estimated to 3.4 ± 0.11 nM (Fig. 2e).

### In vivo rodent PET studies

#### Distribution of [^68^Ga]ABY-028 with the plasma pool

**Fig. 3:**
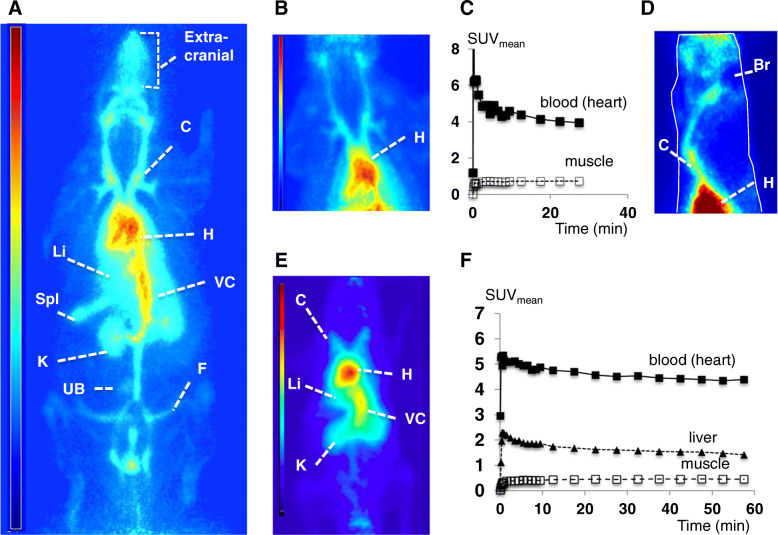
In vivo distribution of radioactivity after i.v. injection of [^68^Ga]ABY-028. **a** Coronal view of the whole body of a Sprague-Dawley rat; constant bed motion acquisition during 0–45 min. **b** Coronal view of the thorax of a NIH-Foxn1mu rat; fixed bed acquisition 0–30 min. **c** Time activity curves (TACs) of the mean standard uptake values (SUV_mean_) from regions in the rat in **b**. **d** Sagittal view of the head, neck, and upper thorax of the rat in **b**; fixed bed acquisition during 34–64 min. **e** Coronal view of the whole body of a SCID mouse; fixed bed acquisition during 0–60 min. **f** TACs from regions in the mouse in **e**. Br, brain; C, right jugular vein ± common carotid; F, femoral vessel; H, heart; K, kidney; Li, liver; Spl, spleen; UB, urinary bladder; VC, vena cava ± aorta

The patterns of radioactivity distribution observed in vivo after the i.v. injection of [^68^Ga]ABY-028 in rats and mice were consistent with a rapid and persistent binding of the radiotracer with serum albumin and distribution with the plasma pool (Fig. 3). Most of the radioactivity remained in the circulatory system throughout observation periods up to 180 min, with highest levels in ventricles of the heart and the major vessels throughout the body (Fig. 3a, b, d, e). The slow decrease of blood radioactivity (8% decrease from 7 to 30 min (rat, Fig. 3c) and from 7 to 60 min (mouse, Fig. 3f) and the small but measurable uptake in muscle tissue are consistent with gradual transport from the circulatory system to tissue interstitium and gradual systemic elimination. Radioactivity was observed in the blood-rich liver and spleen (Fig. 3a). Radioactivity concentrations in the lungs of the rodents could not be resolved due to spillover from the very large concentrations in the heart. There was radioactivity in extracranial tissues and vessels but not in the healthy rat brain (Fig. 3d). Dynamic scans performed over almost the whole body of mice (Fig. 3e) revealed distribution patterns consistent with those observed in the rat. In spite of the poorer resolution of small mouse organs when imaged with ^68^Ga, the time activity curves obtained for the ventricles of the heart and muscle were similar to those observed in the rat and the activity in the liver followed the same slow decline consistent with that of the plasma pool (Fig. 3f). The whole body PET studies of [^68^Ga]ABY-028 revealed no quantifiable radioactivity concentrations in the intestines, independent of the scaling, while radioactivity concentrations in the urinary bladder increased over time.

#### Uptake of [^68^Ga]ABY-028 in experimental tumors

The uptake of [^68^Ga]ABY-028 was examined in several rodent tumor models. First, in initial pilot studies, xenograft models in mice (lung, breast, prostate, head and neck cancer) were imaged with [^68^Ga]ABY-028 (data not presented here). The levels of radioactivity uptake in tumors varied between models, but in most cases, the xenografts were located too close to host blood vessels that contained large concentrations of radioactivity. Spillover effects made quantifications of the small lesions unreliable and, consequently, the xenograft studies were thereafter performed in rats.

**Fig. 4:**
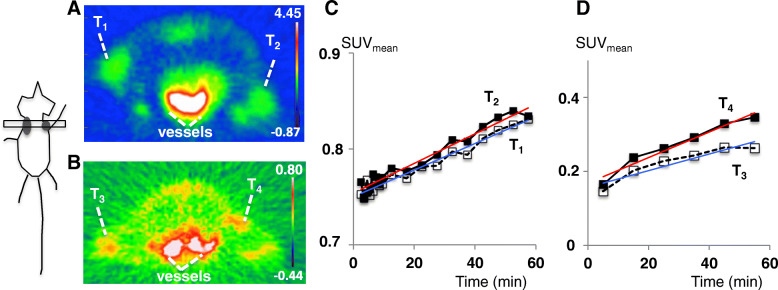
Differing uptake of albumin-bound [^68^Ga]ABY-028 in two xenograft tumor models in NIH-Foxn1mu rats. Both images are summed over 60 min from tracer injection. The scheme to the left shows the approximate location of the xenografts. The dynamic ranges of the SUV_mean_ scales for each image are shown in the color bars to the right. Transaxial PET images are **a** the two FaDu xenografts T_1_ and T_2_ and **b** the two A431 xenografts T_3_ and T_4_. TACs are for **c** FaDu tumors T_1_ and T_2_ and for **d** A431 tumors T_3_ and T_4_. The *y*-axes are different in the two graphs to facilitate visual comparisons between the uptakes in each of the two tumors. Fitted lines are drawn in red and blue

Second, uptakes in two human tumor xenograft models (FaDu or A431) in rats were compared (Fig. 4a, b). Dynamic imaging following the i.v. injection of [^68^Ga]ABY-028 revealed pronounced differences in the initial radioactivity uptake in the xenografts and the differences between the models changed over time. At 5 min, the uptake was approximately 5 times higher in the FaDu than in the A431 tumors (SUV_mean_ 0.768 and 0.754 for T_1_ and T_2_ vs. 0.145 and 0.165 for T_3_ and T_4_, respectively), consistent with a higher vascular volume in the FaDu tumors. The radioactivity levels in both xenograft models increased steadily during the imaging period (Fig. 4c, d). The rates of increase in uptake in the two FaDu xenografts were similar (slopes 0.0014 and 0.0015 for the fitted lines for T_1_ and T_2_, respectively). In the two A431 xenografts, the rates of increase were larger than in FaDu and were different from each other. The greater increase rate was observed in the larger tumor (slopes 0.0022 and 0.0035 for T_3_ and T_4_, respectively). At 60 min, the differences in uptake between the two models had decreased: the uptake in FaDu was now 2.5–3 times higher than in A431 (SUV_mean_ 0.83 and 0.83 for T_1_ and T_2_ vs. 0.26 and 0.35 for T_3_ and T_4_, respectively).

**Fig. 5:**
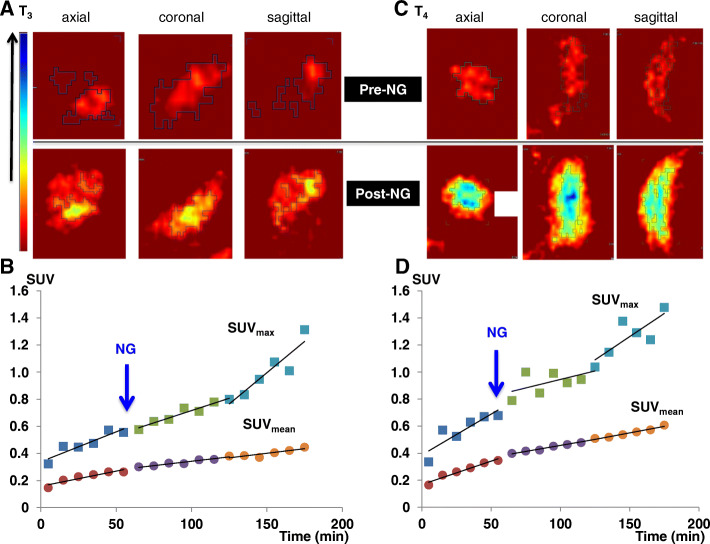
Nitroglycerin (NG) effect on the uptake of albumin-bound [^68^Ga]ABY-028 in A431 xenografts. **a**, **c** Axial, coronal, and sagittal PET images of **a** T_3_ and **c** T_4_. Upper row (pre-NG) images are summed over 0–60 min following the tracer injection, lower row (post-NG) images are summed over 120-180 min. All images use the same scale of SUVs shown in the color bar to the left. **b**, **d** Mean and maximum SUV TACs in T_3_ and T_4_, respectively, for 60 min prior to and 120 min after topical application of NG show that NG-induced changes are revealed by the SUV_max_

To examine whether [^68^Ga]ABY-028 uptake is sensitive to (and might be useful to monitor) manipulations in the tumor vascular environment, vasodilating NG ointment was applied topically just above the A431 xenografts in the rat shown in Fig. 4b at the end of the first hour of imaging and scanning was continued (Fig. 5). During the first hour of imaging (Fig. 5a, c, pre-NG images), the uptake of radioactivity was low and primarily in small central areas of the tumors. After NG had been administered (Fig. 5a , c, post-NG images), the uptake continued to spread throughout a larger area in both tumors and the mean radioactivity uptake continued to increase (SUV_mean_ 0.45 and 0.61 for T_3_ and T_4_, respectively, at 180 min, Fig. 5b, d). A more rapid increase in focal radioactivity levels, revealed by the SUV_max_ time activity curves (TACs) (Fig. 5b, d), started at 1 h after the NG administration and continued during the rest of the scan (SUV_max_ 1.31 and 1.48 for T_3_ and T_4_, respectively, at 180 min, Fig. 5b, d). Interestingly, the SUV_mean_ of both tumors had after 3 h still not reached the initial uptake levels of the FaDu xenografts in Fig. 4, i.e., uptake differences between the two models were maintained during this observation period.

**Fig. 6:**
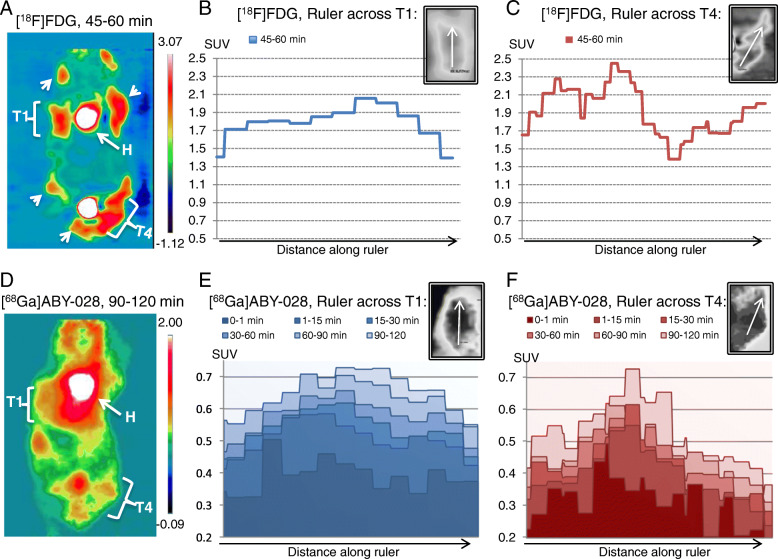
Comparison of metabolism and permeability in a MMTV-PyMT mouse with multiple mammary tumors, two of these depicted here by the brackets and as T1 and T4, arrowheads showing other tumors. **a**, **d** Coronal PET images of the radioactivity uptake after i.v. injection of [^18^F]FDG and [^68^Ga]ABY-028, respectively. The different scales of the SUVs in the images are shown in the color bars to the right of each image. **b**, **c**, **e**, **f** Profiles of radioactivity concentrations along a ruler placed at one position (placements shown in the gray inserts above each graph) in T1 and T4. Only one graph for [^18^F]FDG uptake is shown in **b** (T1) and **c** (T2) since the peaks and profiles did not change appreciably after the early uptake phase. With [^68^Ga]ABY-028 in **e** and **f** the 0–1 min profile indicated the vascular radioactivity while the subsequent profiles visualized the changes in the degree and local heterogeneity of the leakage of the albumin-bound radioactivity into parts of the tumors over time

Third, following the observation that the uptake of [^68^Ga]ABY-028 differed in the two A431 tumors (Fig. 4b), a study was also performed in a MMTV-PyMT transgenic mouse with multiple mammary tumors of differing vascular permeability, some of which were not located too close to major vessels (Fig. 6). Due to the resolution concerns in the mouse, scanning was first performed with [^18^F]FDG to better define the tumor areas and then with [^68^Ga]ABY-028 on the following day after the ^18^F had decayed. High [^18^F]FDG uptake was observed in the cancerous mammary glands (two in the coronal slice in Fig. 6a are marked by brackets). The corresponding uptake of albumin-bound [^68^Ga]ABY-028 was lower (1/4 to 1/3 that of [^18^F]FDG after 2 h) and, as observed in the preliminary xenograft studies in mice, some of tumors were not resolvable with this tracer when the mammary glands were too close to the large radioactivity in the heart and large vessels. Two of the tumors that had high uptakes of both tracers in the same coronal slice are shown here (T1 and T4 in Fig. 6a, d). An IRW ruler was drawn through the middle of the tumor and summation images were generated for different time frames to show the patterns of local radioactivity build-up in the tumors. For the [^18^F]FDG studies, the profiles were essentially identical in images summed over 30–45 min and 45–60 min in both tumors and therefore only the latter was shown here. The radioactivity concentrations along the ruler through tumor T1 (Fig. 6b) were fairly homogenous and, except at the very edges, SUVs varied at most by 15%, from 1.7 to 2.05. In the tumor marked T4, however, the line profile (Fig. 6c) showed a heterogeneous [^18^F]FDG uptake, with SUVs varying from as high as 2.45 to as low as 1.38. Profiles of the albumin-bound [^68^Ga]ABY-028 in both tumors in Fig. 6e, f showed that radioactivity concentrations continued to increase throughout the observation period (2 h). In tumor T1, the pattern of the profile at 90–120 min was similar to that of the glucose utilization in Fig. 6b. Interestingly, during the first 30 min, permeability appeared to be higher in the part of the tumor that had lower final uptake of [^18^F]FDG (to the left in the line profile and at the bottom of the tumor). In tumor T4, the uptake of the albumin-bound [^68^Ga]ABY-028 (Fig. 6f) was, similar to the [^18^F]FDG profile, much more heterogeneous than in tumor T1. The areas of the tumor with the highest glucose utilization had lower uptakes of the [^68^Ga]ABY-028 radioactivity than were observed in T1. Although exact correspondence between placement of the tumors in the two different experiments was impossible, both visual assessment and observations of the line profile changes by varying the placement of the ruler across T4 strongly indicated that the area with highest albumin-bound [^68^Ga]ABY-028 was close to the area in the middle of the tumor where the [^18^F]FDG uptake was at its lowest, i.e., an inverse relationship between the metabolic activity of the tumor and the permeability to the albumin-bound protein [^68^Ga]ABY-028.

**Fig. 7:**
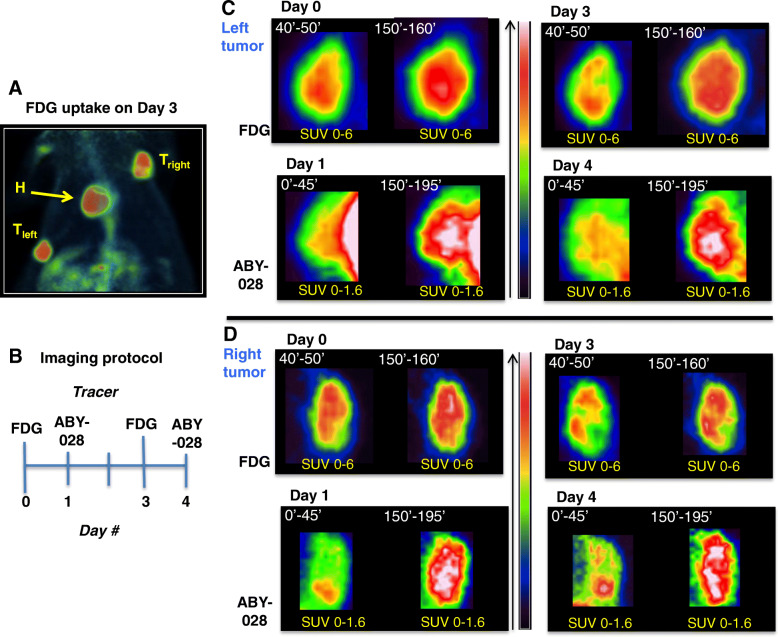
Comparison of changes in metabolism and permeability with tumor progression in a NIH-Foxn1mu rat bearing two FaDu xenografts. **a** Maximum intensity projection PET image of [^18^F]FDG uptake on day 3 showing the localization of the xenograft tumors (T) to the left and right; H, heart. **b** The time line for imaging experiments. **c**, **d** Coronal PET images for the left and right tumors, respectively: the top rows are of [^18^F]FDG uptake on day 0 and day 3, dual time imaging on each day, and the bottom rows are of [^68^Ga]ABY-028 uptake on day 1 and day 4, dual time imaging on each day, but at different times for the two tracers (shown immediately above each image). The same magnification was used in the images. The bar shows the color scale used. Note: to facilitate visual comparisons the SUV_mean_, ranges in the color bar are the same for all the [^18^F]FDG images (0–6) and for all the [^68^Ga]ABY-028 (0–1.6), but are different for the two tracers due to their differing levels of total uptake

To demonstrate whether the albumin-bound tracer might be used to visualize permeability changes over time within a tumor, a rat with two FaDu xenografts was scanned using [^18^F]FDG and [^68^Ga]ABY-028, twice with each tracer, 3 days apart (Fig. 7). The xenograft placements are shown in the [^18^F]FDG image in Fig. 7a and the study design protocol in Fig. 7b. Images were generated for two time periods after the i.v. injection (given in the upper left corner of the images in Fig. 7c, d) since dual time point imaging of [^18^F]FDG in tumors has been used to improve detection sensitivity and differentiation from inflammation and normal tissue [44, 45]. Here, the overall [^18^F]FDG uptake increased between the early and late imaging time points by approximately 20% and 35% on days 0 and 3 in both tumors but uptake patterns were mainly unchanged (Fig. 7c, d, upper rows). The corresponding multiple-time imaging with the albumin-bound [^68^Ga]ABY-028 indicate heterogeneity in permeability and increases in uptake as the tumor grew:

On days 0/1: In the *left* tumor (Fig. 7c, first two sets of images), the [^18^F]FDG uptake was fairly homogenous after both 50 and 160 min, with highest SUVs in the middle and a gradual decrease toward the periphery. Only very low levels of albumin-bound [^68^Ga]ABY-028 were in the tumor during the first 45 min—so low and so close to blood-rich host tissue that the tumor was hardly distinguishable. By 3 h, however, the albumin-bound [^68^Ga]ABY-028 had spread throughout most of the tumor. The levels in the central part were approximately 40% higher than in the periphery. In the *right* tumor (Fig. 7d, first two sets of images), the uptake of both tracers is much more heterogeneous at both scan times. This tumor is larger than the left tumor and the areas of hypometabolism at the lower edge could be indicative of necrosis. Similar to the observations in the MMTV-PyMT mouse, the area with the lowest [^18^F]FDG uptake is that in which the early uptake of the albumin-bound [^68^Ga]ABY-028 is largest. By 3 h, the protein had spread throughout the tumor, though highest concentrations were still in the lower part of the tumor. In both the left and right tumors, dual time point imaging with albumin-bound [^68^Ga]ABY-028 clearly revealed local differences in tracer uptake that were not as evident with [^18^F]FDG.

On days 3/4: In the *left* tumor (Fig. 7c, second two sets of images), an area with decreased [^18^F]FDG uptake (approximately 30% lower SUV) was observed in the center and extending to one edge of the tumor. This is true for both time points, though it is not quite as visible here in the 160 min image (since the color scale settings were the same). In contrast, the albumin-bound [^68^Ga]ABY-028 uptake is clearly highest in the center of the tumor where the metabolism is lowest and is much higher in the 45 min image than it was on day 1. The same trend is true for the *right* tumor (Fig. 7d, second two sets of images). The patterns observed in both tumors are consistent with the development of local vascular disruptions and increases in permeability to albumin as parts of the tumor become less metabolically viable (hypoxic/necrotic) over time.

#### Uptake of [^68^Ga]ABY-028 after acute induced ischemic damage to the BBB

**Fig. 8:**
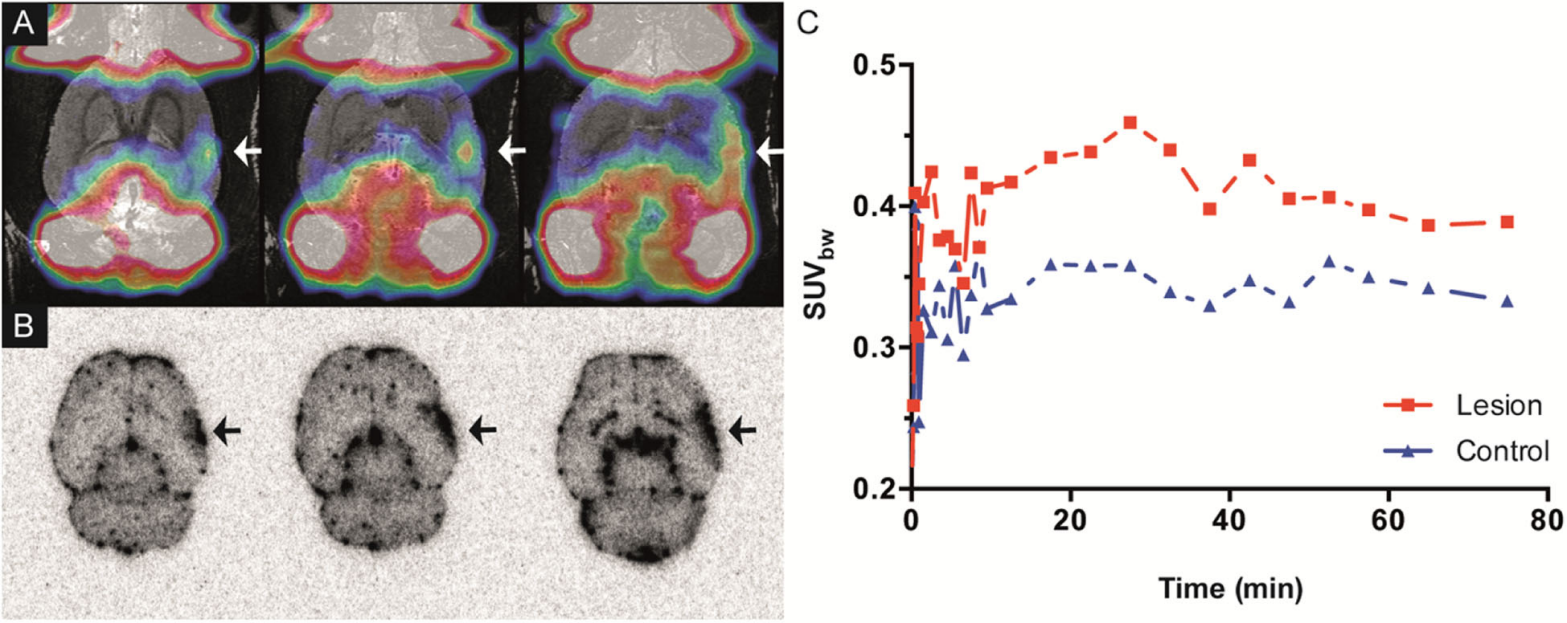
Increased permeability in the acute phase after an induced focal cortical infarction. **a** Three successive axial PET images of the uptake of albumin-bound [^68^Ga]ABY-028-PET in the infarcted (arrowheads) rat brain summed over 0-80 min fused with a magnetic resonance image of a healthy rat. **b** Ex vivo phosphorimages of corresponding sections from the rat that was imaged with [^68^Ga]ABY-028. **c** TACs of radioactivity uptake in the infarcted lesion and the contralateral control ROI

To illustrate the potential use of albumin-bound [^68^Ga]ABY-028 to delineate acute disruptions of vascular permeability in small cerebral lesions and predict the cerebral transport of ABD-based therapeutics, we used a model in which a focal cortical infarction was induced in a rat by placing a microwire in the MCA [39] immediately prior to and remaining for 80 min after the radiotracer administration (Fig. 8). This model has been shown to produce mean infarct volumes of 23 mm^3^. This is ≈ 8% of the cerebral hemisphere of the rat and similar in relative sizes to those found in strokes in humans, but is close to detection limits for small animal imaging even though background uptake in areas with intact BBB should be low. Therefore, the in vivo results were corroborated by higher resolution ex vivo phosphorimaging of the excised brain immediately following the PET scan. An area with increased uptake of albumin-bound [^68^Ga]ABY-028 extending through three successive PET images summed over the entire 80 min was detected (Fig. 8a). Fusion with a reference MRI image of a healthy rat and the ex vivo PI images (Fig. 8b) confirmed the localization of an infarct to the expected cortical area. Though quantification in a lesion of this size is impeded by partial volume effects, the radioactivity uptake in the infarcted ROI was clearly larger and different from that in the contralateral side, which mainly was radioactivity in the cerebral vessels (Fig. 8c). Leakage through the BBB apparently occurred immediately after the damage. The leakage increased during the first 30 min and thereafter decreased with a tendency to leveling off. Radioactivity in the contralateral ROI was, within statistical variations, essentially the same throughout the scan.

## Discussion

The albumin binding capability of ABD-based proteins is utilized to prolong their in vivo circulatory half-lives and to improve their uptakes in tissues with a compromised endothelium. Here, we have successfully labeled the ABD-based ABY-028 with ^68^Ga to obtain a short-lived positron emitting tracer that distributes with albumin so the vascular permeability to albumin-borne ABD-based and similar sized therapeutics can be assessed. We have illustrated in rodent models that this PET radiotracer can be useful for visualizing heterogeneities in altered vascular permeability to albumin in the early phase of diseases, as lesions change over time and after pharmacological interventions.

Synthesis of the crude ABD peptide followed by site specific coupling of mal-DOTA and purification yielded the active, i.e., albumin binding, product ABY-028 with high purity. Labeling ABY-028 using the fractionated ^68^Ga generator eluent was efficient. Since albumin was not used during the radiolabeling, possible risks from handling blood components were avoided. The addition of EDTA after labeling followed by the SPE purification gave a product of high radiochemical purity. The in vitro study showed that ABY-028 retained binding to HSA after ^68^Ga labeling and that its binding could be blocked by the unlabeled peptide, which indicates that binding of the labeled and unlabeled peptides occur at the same sites (Fig. 2d). The measured affinity of 3.4 nM was lower (i.e., a higher value) than that previously reported for the ABD protein [19]. This could be due to the methods used and that only a 1 h incubation time (with considerations to the half-life of the radionuclide) was used, which may be too short to reach equilibrium. The results obtained are, however, interpreted as clear indications that the labeling did not destroy the albumin binding capability of ABY-028.

Studies in the healthy rat (Fig. 3a, b, d) showed that the radioactivity patterns are consistent with those reported using smaller albumin-binding molecules (e.g., [18]). This provides further support that the labeled ABY-028 binds in vivo to albumin, thereby acquiring its circulatory behavior. The liver and spleen were clearly visualized since they are well-perfused organs with fenestrae large enough to be permeable to albumin. Uptake in the normal brain was not detected nor expected since the blood-brain barrier was intact. Abnormal depositions in the lungs indicative of labeled colloids or aggregates were not observed. That radioactivity levels gradually decreased in the blood and gradually increased in well-vascularized muscle tissues is consistent with the normal extravasation behavior for albumin. The gradual elimination of radioactivity through the kidneys to the bladder is consistent with the renal degradation and elimination of albumin [46]. The distribution in the mouse (Fig. 3e) was comparable to that in the rats, though resolution of its smaller structures is difficult and thus the quantifications of the radioactivity concentrations should be considered less reliable.

The high E_β+max_ of ^68^Ga reduces the intrinsic spatial resolution [47], which becomes an increasing concern as the size of the animal imaged is decreased. Most of these studies were performed in rats instead of mice to minimize as much as possible spill-over effects on quantification [48]. ^68^Ga-Labeled tracers are, of course, very successfully used in clinical imaging (e.g., [49, 50]). ^68^Ga was chosen as the radionuclide for the ease of labeling and because it would have sufficient decays for reasonable statistics during the initial tracer distribution, but still be long-lived enough for imaging after several hours. During the first few minutes after administration, large macromolecules reside mostly in the vessels and images from that time period should primarily reflect tissue vascularization. As extravasation begins, the transport of a large tracer should be essentially unidirectional. How and where the radioactivity is taken up during this phase should indicate local variations in vessel permeability. It is more optimal to use a short-lived radiotracer to study this part of the deposition of the imaging ABD-based protein. At late times, breakdown of the tracer-albumin interaction, albumin and the tracer molecule, behavior of the metabolites and (dys)functions of the lymphatic system will all contribute to how much radioactivity is retained in the tissue. The slow clearance of radioactivity from the blood after the first 15 min (Fig. 3c, f) and the steadily increasing radioactivity concentrations observed in the diseased tissues (Figs. 4, 5, and 6) are consistent with high in vivo stability of the radiotracer complex with albumin. A longer-lived radiotracer would be desirable for examining net effects due to specific retentions of the tracer and possible metabolites and to EPR mechanisms. The DOTA chelator used here allows for complexation with ^89^Zr or ^111^In for imaging with PET or SPECT, respectively, for later windows of observation.

Since extravasations of a macromolecular therapeutic or imaging agent in tumors will vary with the vessel leakiness (e.g., [51]) and with time, it is important to take into account variations in blood vessel leakiness, histological grades, and malignant potential [52] in the models used. Examples of the ways these variations can impact on the depositions of the theranostics were illustrated here using two xenograft models, FaDu and A431. FaDu is an aggressive tumor with a high proliferation rate [53] and a rapidly increasing necrotic fraction [54]. The initial uptake of albumin-bound [^68^Ga]ABY-028 in the two FaDu xenografts was fast and large (Fig. 4a, c), which is consistent with its known high permeability and high intravascular volumes (the fluid influx that occurs early in the development of necrosis) [55]. A431 xenografts are known to be highly angiogenic and grow rapidly, but are homogenously vascularized with smaller, not extensively branched vessels [56]. Initial uptake of albumin-bound [^68^Ga]ABY-028 in A431 tumors was much lower than in the FaDu tumors, but the subsequent rate of increase was nearly twice as high (consistent with angiogenic and but less necrotic and very vascularized tissues) (Fig. 4b, d). The more reliable imaging of variations in vascular properties at early times is consistent with previous findings that ^68^Ga-DOTA-albumin uptake revealed larger uptakes in highly angiogenic hepatomas than in the wild type tumors at 10–15 min [57]. It is also consistent with PET studies that revealed small differences in ^89^Zr-DFO-albumin uptake in three xenograft models of prostate cancer at 1 h but not at 20 h, despite marked histological differences [2]. *Intra*-tumoral differences in vessel leakiness were also visualized here by the *early* uptake of the albumin-bound [^68^Ga]ABY-028 in spontaneous tumors (Fig. 6e, f) as well as in xenografts (Fig. 7c, d, 0–45 min vs. 150–196 min). Passive uptakes within a tumor varied by up to a factor of two. This definitely argues against analyzing tumor tissues as a single ROI and argues for, at a minimum, segmenting out sub-regions according to their heterogenoeus vascular properties. The time span over which tumor models are used is also important since vascular properties will most probably change. This is clearly observable in the changes in the *early* uptake of the albumin-bound [^68^Ga]ABY-028 in the two untreated FaDu xenografts in Fig. 7c, d (day 1 vs. day 4).

In the MMTV-PyMT and FaDu tumors (Figs. 6 and 7), sub-regions within the tumor demonstrated inverse relations between glucose utilization measured with [^18^F]FDG and the uptake of albumin-bound [^68^Ga]ABY-028. Low [^18^F]FDG/high [^68^Ga]ABY-028 uptake at early imaging times is interpreted to indicate more necrotic areas in which metabolic activity has decreased and the uptake of fluids has increased as cells begin to die. Conversely, high [^18^F]FDG/low [^68^Ga]ABY-028 uptake at early imaging times would instead indicate viable and/or prenecrotic tumor [58]. Increased glucose consumption is one of the hallmarks of cancer [59]. Highly proliferating tumor cells recruit and grow along tumor vessels to ensure their supply of nutrients and oxygen. The blood vessels are leaky but the sheaths of cells and high intra-tumor pressure can hinder penetration of macromolecules [60]. This could explain the low uptake of albumin-bound [^68^Ga]ABY-028 in the higher metabolic areas and increasing uptake as permeability increases with progressing cell death. This kind of non-invasive viability analysis in sub-regions of intra-tumor heterogeneity would be important for correct differentiations, but also for predicting to what degree and when similar size therapeutics would reach the more viable parts of the tumor. Future studies to further examine the basis of this metabolism/permeability mismatch, e.g., by additional in vivo necrosis imaging protocols or by ex vivo correlations of radiotracer uptake to histopathological markers, are warranted.

The detachment of tumor cells and entrance into the circulatory system through leaky tumor vessels is an important part of the metastatic cascade [61]. Characterization of the vascular permeability of tumors (their metastatic potential) could be very useful for predicting the potential and monitoring the effect of therapeutics to suppress that vascular permeability. In the transgenic MMTV-PyMT model, multiple tumors develop spontaneously and simultaneously within the mammary glands of the mouse and spontaneous metastasis to the lungs occurs in most animals by 12–13 weeks [62, 63]. Uptake of albumin-bound [^68^Ga]ABY-028 was observed here in all the tumors in a 12-week old mouse, at the age for metastatic spread (Fig. 6). The heterogeneity of the vascularity within and between the PyMT tumors is clearly visualized in the early radioactivity profiles of the two tumors in Fig. 6e, f. Both a higher level of uptake and a more rapid rate of increase of albumin-bound [^68^Ga]ABY-028 in T1 than in T4 are indicative of a later progression. This model together with longitudinal [^68^Ga]ABY-028 PET studies could be appropriate for studying permeability changes as spontaneous mammary tumors progress from normal to invasive stages and also the effects of therapeutics on that permeability.

Considerable research is being performed on physically or pharmacologically modulating the physiological state of tumors to improve the delivery of (large) therapeutics [64]. NG has been used to modulate the EPR-driven uptake of macromolecular drugs in tumors [65]. We demonstrated here increased uptake of albumin-bound [^68^Ga]ABY-028 occurred at times consistent with the previously reported onset of vasodilatory actions of NG [65]. In spite of the acknowledged difficulties in characterizing images using texture analyses even in human tumor tissues [66], the heterogeneity in our [^68^Ga]ABY-028 images suggests that those methods might improve evaluations of the dynamics and effectiveness of local vascular effects. We suggest that changes induced in the early uptake of albumin-bound [^68^Ga]ABY-028 could be used to monitor the efficacy of therapeutics that alter the vascular environment. A method separating potentially varying blood volumes and permeabilities such as that used in studies with [^18^F]FAl-NEB [67] could be valuable, especially in investigations of pharmacological interventions.

Albumin is proposed as a carrier for diagnostics and therapeutics that target other diseases than cancer that have altered vascular permeability such as degenerative neurological disorders. In acute ischemic stroke, cytotoxic and vasogenic edema occur and are accompanied by leakage of serum macromolecules [68]. We used a model of acute focal occlusion to examine the feasibility of detecting uptakes of serum albumin-bound ABD-based probes in small diseased cerebral tissues. Albumin-bound [^68^Ga]ABY-028 leaked into the lesion (Fig. 8) at levels that were higher than those in the contralateral side throughout the 1 h scan. The time of maximum uptake and the subsequent decrease as well as a residual retention of radioactivity were all detected, the latter of which was confirmed by ex vivo autoradiography at 80 min after the start of the occlusion. The pathogenesis of edema development in stroke will differ over time and for each individual. The need for assessing permeability in stroke has been recognized and dynamic contrast-enhanced MRI and CT are currently the modalities most used clinically [69]. Nuclear medicine methods and in particular PET have, however, played and still have a role to play in research on the pathology of stroke and on the development of new therapeutics [70]. Due to the molecular sensitivity of PET, probe microdosing minimizes risks for toxic reactions and/or other perturbations on the targeted or non-targeted tissues, particularly for repeated (longitudinal) studies. PET imaging with albumin-bound [^68^Ga]ABY-028 might be a useful tool for identifying the time window for the acute cerebral leakage of albumin, for studying the ability of anti-vasogenic edema drugs to prevent abnormal leakage of albumin and similar size substances into the brain [71] and for predicting the probability that albumin-ABD-based therapeutics can enter the brain in this and potentially also in other neurological disorders such as epilepsy, Alzheimer’s disease, Parkinson’s disease, multiple sclerosis, dementia, traumatic brain injury, and inflammation.

As discussed above, the distribution patterns of the radioactivity in healthy animals are consistent with those of other radiotracers that also bind in vivo with high affinity to and are slow to release from albumin. A direct comparison to the behavior of these other albumin-binding radiotracers in animal models is not possible since the study protocols used and the models studied here are not the same and our studies indicate that uptake of this type of tracer in diseased tissue is highly individual. However, the following advantages and disadvantages of [^68^Ga]ABY-028 are noted: ^68^Ga is attractive since it is readily available for facile radiolabelings and is an appropriate radiolabel for permeability studies of 2–3 h durations and for consecutive paired radioimaging studies. Conversely, if long-term net uptakes requiring longer observation times are needed, longer-lived radionuclides such as ^89^Zr or ^111^In should be used. For imaging very small structures such as lymph nodes, ^18^F or the longer-lived radionuclides instead of ^68^Ga would be more favorable. [^68^Ga]ABY-028 is the best tool for, in paired in vivo studies, probing the degree of non-specific uptake of ABD-based radiodiagnostics in tissues with altered permeability. [^68^Ga]ABY-028 is also the best tool for, in non-paired imaging studies, predicting the probability of delivering related ABD-based therapeutics to the diseased tissues.

## Conclusions

This study has demonstrated that [^68^Ga]ABY-028 distributes in vivo in a fashion that is consistent with its rapid in vivo binding with plasma albumin. The albumin-bound ABD-based protein revealed, in early in vivo mapping, heterogeneities in the uptake in different sub-regions of the same tumor, between different tumor models, over time as well as in response to a vasodilatory challenge. It was also possible to follow disease-related leakage of the albumin-bound ABD-based radiotracer in small lesions in the brain. [^68^Ga]ABY-028-PET offers a non-invasive technique for characterizing tissue permeability to albumin bound ABD-based (and similar-sized) macromolecular therapeutics at baseline, over time and potentially during permeability-altering therapeutic interventions.

## Data Availability

Requests for the datasets used in the current study should be made to the corresponding author. Requests for ABY-028 should be made to Affibody AB.

## Abbreviations

ABD: Albumin-binding domain
BBB: Blood-brain-barrier
CBM: Constant bed motion
CH_3_CN: Acetonitrile
DCM: Dichloromethane
DMF: Dimethylformamide
DP: Drug product
DS: Drug substance
EDTA: Ethylenediaminetetraacetic acid
EtOH: Ethanol
FcRn: Neonatal Fc receptor
[^18^F]FDG: [2-^18^F]-2-fluoro-2-deoxy-D-glucose
^68^Ga: Gallium-68
GMP: Good manufacturing practice
HLB: Hydrophilic-lipophilic-balanced
H_2_O: Water
HPLC: High-performance liquid chromatography
HSA: Human serum albumin
IPA: Isopropyl alcohol
IRW: Inveon Research Workplace
iTLC: Instant thin layer chromatography
i.v.: Intravenous
Kd: Equilibrium binding constant
MCA: Middle cerebral artery
NaCl: Sodium chloride
mal-DOTA: 1,4,7,10-Tetraazacyclododecane-1,4,7-tris-acetic acid-10-maleimidoethylacetamide
MMTV: Mouse mammary tumor virus
NFDM: Non-fat dry milk
NG: Nitroglycerin
PBS: Phosphate-buffered saline pH 7.4
PBST: (1× PBS/0.05% Tween)
PET: Positron emission tomography
PyMT: Polyoma middle tumor-antigen
ROI: Region of interest
RPC: Reversed phase chromatography
RT: Room temperature
s.c.: Subcutaneous
SCID: Severe combined immunodeficiency
SEC: Size exclusion chromatography
SPE: Solid phase extraction
SPECT: Single-photon emission computed tomography
SPPS: Solid phase peptide synthesis
SUV: Standard uptake values
TFA: Trifluoroacetic acid
